# Managing Encapsulated Oil Extract of Date Seed Waste for High Hydroxyl Radical Scavenging Assayed via Hybrid Photo-Mediated/Spectrofluorimetric Probing

**DOI:** 10.3390/molecules28135160

**Published:** 2023-07-01

**Authors:** Amr A. Essawy, Khaled F. El-Massry, Ibrahim Hotan Alsohaimi, A. El-Ghorab

**Affiliations:** 1 Chemistry Department, College of Science, Jouf University, Sakaka 72388, Saudi Arabia; ehalshaimi@ju.edu.sa (I.H.A.); aghorab@ju.edu.sa (A.E.-G.); 2 Chemistry Department, Faculty of Science, Fayoum University, Fayoum 63514, Egypt; 3 Flavour and Aroma Chemistry Department, National Research Centre, Cairo 12622, Egypt; kfaroak@ju.edu.sa

**Keywords:** nano-titania, solar productivity of hydroxyl radical, antioxidant potential, encapsulated date seed oil, fluorimetric probing

## Abstract

This work addresses two research topics: the first concerns the specific/sensitive trapping of hydroxyl radicals (^•^OH), and the second concerns the efficacy of encapsulating natural antioxidants, potentially lengthening their preservation activity. For context, nano-titania was solar-irradiated to produce ^•^OH, which was spectrofluorimetrically assessed, based on the selective aromatic hydroxylation of the non-fluorescent sodium terephthalate to 2-hydroxyterephthalate fluorophore. Fluorescence intensity is proportional to generated ^•^OH. Thus, a simple/rapid indirect method was utilized to assess ^•^OH precisely. Accordingly, novel photoluminescent system is outlined in order to assess the scavenging potentiality of ^•^OH in date seed oil (DSO) in both its pure and encapsulated formulations (ECP–DSO), i.e., when fresh and 5 months after extraction and encapsulation, respectively. With the addition of 80 μg/mL DSO or ECP–DSO, the efficacy of ^•^OH scavenging amounted to 25.12 and 63.39%, which increased to 68.65 and 92.72% when 200 μg/mL DSO or ECP–DSO, respectively, was added. Moreover, the IC50 of DSO and ECP–DSO is 136.6 and 62.1 µg/mL, respectively. Furthermore, DSO and ECP–DSO decreased the kinetics for producing ^•^OH by ≈20 and 40%, respectively, relative to ^•^OH generated in the absence of antioxidant. This demonstrates the benefits of encapsulation on the preservation activity of natural antioxidants, even after five months after extraction, in terms of its interesting activity when compared to synthetic antioxidants. The developed fluorimetric ^•^OH probing upgrades antioxidant medicines, thus paving the way for theoretical/practical insights on mechanistic hydroxyl radical-damaging biology.

## 1. Introduction

Reactive free radicals have attracted more attention because of their significant roles in countless physiological and pathological processes in the chemical, biological, and medical fields. They have been involved in the development of many diseases due to increased oxidative stress, such as aging, cancer, coronary artery disease, autoimmune disease, diabetes, sclerosis, atherosclerosis, cataract, chronic inflammation, and cellular signaling [1]. Free radicals caused impairment, which is ascribed to their ability to deactivate many cells (protein denaturation, membrane destabilization, DNA mutation, and lipid peroxidation) [2,3,4].

In all aerobic organisms, oxidative stress occurs when the reactive oxygen species (ROS) produced along with peroxides (H_2_O_2_), hydroxyl (^•^OH), and other radicals surpass the inadequate cellular antioxidant system capacity, giving rise an oxidative phosphorylation producing ROS. Among free radicals, the short-lived ^•^OH radical is the most reactive, aggressive, and particularly dangerous species towards a variety of biological species [5]. In photocatalytic reactions, the photoexcitation of semiconductors generates holes (h^+^) that convert H_2_O molecules or ^−^OH ions into ^•^OH radicals [6,7,8]. In cells, the catalyzed decomposition of H_2_O_2_ in presence of Fe^2+^ or Cu^+^ is able to generate ^•^OH radicals. ^•^OH has been proven to damage DNA bases, mediating the redox alteration of cell-membrane Ca^2+^ channels, cellular disorders, and finally cells apoptosis.

Many benefits are associated with fruit and vegetable consumption, such as antimicrobial, antibiotic activities, anti-inflammatory, antimutagenic, anticarcinogenic, antithrombotic, and neuro-protective properties as well as the reduction in cholesterol and cardiovascular diseases [9,10,11]. The protection offered has been ascribed to the existence of dietary antioxidants (polyphenols, flavonoids, ascorbic acid, tocopherols, and carotenoids), which have the ability to donate electrons to oxidants, thus stopping the oxidation chain reaction [12,13]. Recently, developing natural antioxidants as a safe and efficient additives to avoid lipid oxidation, food rancidity, and pharmaceutical products has always encountered the inevitable issue that substituting butylated hydroxyanisole (BHA) and butylated hydroxytoluene (BHT) (synthetic antioxidants) may result in toxicity and demand extraordinary industrial budgets [14].

In various fields, researchers are mostly concerned with the study of nutritious seeds that are rich in lipidic compounds, such as triacylglycerids (at levels above 90%), fatty acids, tocopherols, phospholipids, sphingolipids, sterols and others, as well as being rich in antioxidants [15].

Seeds of date palm fruit (Phoenix dactylifera) are considered the rejected part of the fruit. Despite their richness in valuable bioactive and antioxidant compounds, they are locally used in animal feed and non-caffeinated coffee. In folk remedies, date seeds are listed for the management of diabetes, relief for ague and toothaches, and reducing the risk of cancer and certain cardiovascular diseases. In addition, they strengthen the immune system in order to combat liver and gastrointestinal disorders [16]. Significant economic potential can be achieved when undesired date industry by-products are used for functional foods as raw materials enriched with antioxidants [17].

Date seeds contain a high concentration of oil (5–13%), which is high in phenolic compounds, tocopherols, and phytosterols [18,19,20]. Ghafoor et al. (2022) [18] reported percentage yields using Soxhlet (5.21 ± 0.31), as well as the phytochemicals with antioxidant activity (total phenolic content, 124.27 ± 3.48 mg GAE/100 g, and total flavonoid content, 60.43 ± 0.83 mg QE/100 g) of hexane date seed samples. Additionally, Herchi et al. (2014) [21] stated that certain antioxidants, such as total phenolic, total flavonoid, ascorbic acid, chlorophyll, and carotenoids, were found in different values in date seed oil and date flesh oil, extracted via non-polar solvent (petroleum ether). Moreover, other authors [22] reported that the vitamin, mineral, and fatty acid composition of date seed oil (DSO) makes it valuable for food formulations. Additionally, DSO is an interesting source of important nutrients that have a very positive effect on human health.

Microencapsulation is the process of enclosing an “encapsulate” (the core functional component or sensitive substance) in a “capsule” (a shell composed of wall material) for easier handling or protection. Microencapsulating oily extracts to obtain more secure powdered ingredients for food applications is most commonly and cheaply accomplished through spray drying [23,24]. The efficacy of spray drying has been reported alongside which factors affect the efficiency of the encapsulation process [25].

Diverse approaches for measuring ^•^OH radicals and assaying antioxidant properties have been advocated and implemented. These include electron spin resonance spectroscopy (ESR), UV–Vis spectrophotometry, electrochemical sensing, chromatography, chemiluminescence, and fluorescence spectroscopy, which are based on autoxidation inhibition [26,27,28,29,30,31,32,33,34,35,36]. However, each method has its own limitations, such as sophisticated instrumentation, poor selectivity, operational complexity, low reproducibility, and low sensitivity [37]. For instance, spectrofluorimetry is found to be more consistent in assaying ^•^OH radicals and evaluating antioxidant properties with low cost, simple, selective, sensitive, fast, and good, reliable methodologies [38,39].

The reputation of antioxidants as providing beneficial health effects in fruit and vegetable consumption drove this work to identify, for the first time, a sensitive and selective avenue for addressing the ^•^OH radical scavenging efficacy of date seed oil (DSO) in its pure and encapsulated formulations (ECP–DSO) both when fresh and 5 months after extraction and encapsulation. The microencapsulation of DSO was attempted via spray drying method, using maltodextrin and gum Arabic as encapsulating wall materials. The developed approach employed the solar irradiation of nano-titania suspended in non-fluorescent sodium terephthalate in order to produce ^•^OH radicals that were selectively trapped by terephthalate probe-switching on the fluorescence signal of hydroxy terephthalate. The higher sensitivity of fluorimetric probing enables the discriminative monitoring of a possible alteration in the antioxidant components of DSO during its shelf-life, which may affect ^•^OH radical scavenging activity. Accordingly, the impact of encapsulation could be rapidly and precisely addressed.

## 2. Results and Discussion

### 2.1. Characterization of DSO and ECP–DSO Powder

**Figure 1:**
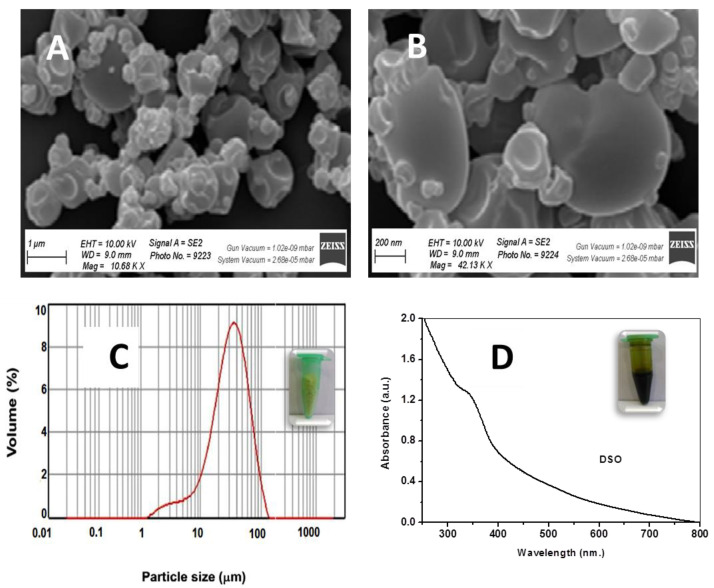
SEM for the morphology of DSO spray-dried powder at two magnifications (**A**,**B**). Particle size of ECP–DSO powder (inset: image of ECP–DSO) (**C**) and the UV–Vis absorption spectrum of DSO (30 μg/mL) (inset: image of DSO) (**D**).

The physicochemical characteristics of DSO and the spray-dried ECP–DSO with maltodextrin (MD):gum Arabic wall material and an efficient encapsulation value (91.28%) were studied. The moisture content was generally within the appropriate range, minimizing the chance of microbial contamination and lipid oxidation [40]. SEM examination revealed no roughness or molded inadequacies on the capsule surface because of slow film formation during the drying of the atomized droplets. Moreover, better spherical homogeneous capsules have smoother surfaces and other larger agglomerates of asymmetrical shapes (Figure 1A,B). Additionally, reconstituted DSO particles in water have a 1–100 μm mean surface diameter (Figure 1C).

### 2.2. UV–Vis Electronic Spectroscopy of DSO

The UV–Vis absorption spectroscopy for the aqueous solution of DSO (30 μg/L) was shown in Figure 1D, where an absorption shoulder is seen at 339 nm and can be attributed to π–π* transitions of phenolics, flavonoids, and anthocyanin compounds. In addition, a noticeable absorption in the visible region was seen in the tailing-off part of the spectrum, which could be attributed to n–π* transitions. The molar absorption coefficient (ε) of the earlier transition gives approximately 1311 L mol^−1^ cm^−1^, while the latter transition gives an estimate of 144 L mol^−1^ cm^−1^.

### 2.3. Characterization of the Nano-Titania

**Figure 2:**
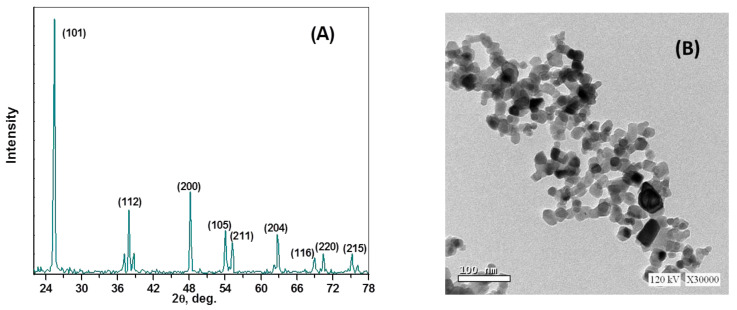
The XRD pattern (**A**) and TEM morphological features (**B**) of the used nano-titania.

The pattern for the XRD of the nano-titania is illustrated in Figure 2A. The diffraction assignments depicted at 2θ = 25.44°, 37.92°, 48.16°, 54.08°, 55.20°, 62.72°, 68.96°, 70.40°, and 75.20° ensures the pure anatase crystallinity for the titania particles with the crystal faces (101), (112), (200), (105), (211), (204), (216), (220), and (215), respectively. Applying the Debye–Scherrer equation (Equation (1)) reveals that the average crystallite size of the nano-titania is estimated to be 14.7 nm.

The investigated morphology was attained using transmission electron microscopy (TEM). As revealed in Figure 2B, the morphology of titania particles increase the predominance of hemispherical/spherical particulation with size estimates ranging from 7.5 to 18.8 nm.

**Figure 3:**
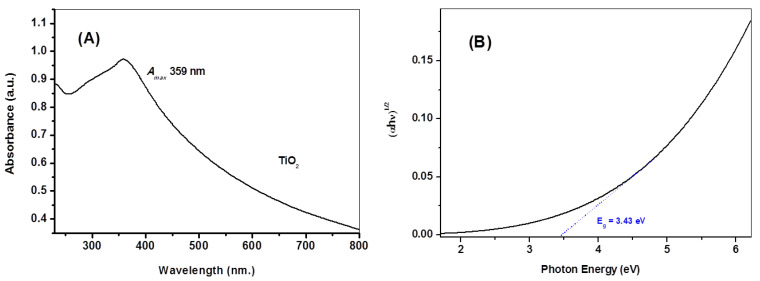
UV–Vis electronic spectra (**A**) and plot of (αhυ)^1/2^ versus hυ of the nano-titania particles (**B**).

Figure 3A illustrates the UV–vis absorption spectrum of the nano-titania particles, where an absorption maximum is seen at 359 nm as a result of surface plasmon resonance and the excitonic absorption of the nano-titania semiconductor, in addition to the considerable tailing-off in the visible range. The plots of the Kubelka–Munk equation (Equation (2)) addresses the band gap (E_g_) of the nano-titania, as depicted in Figure 3B, giving an estimate of 3.43 eV.

### 2.4. Nano-Titania and the Photocatalytic Productivity of ^•^OH Radicals under Solar Irradiation

**Figure 4:**
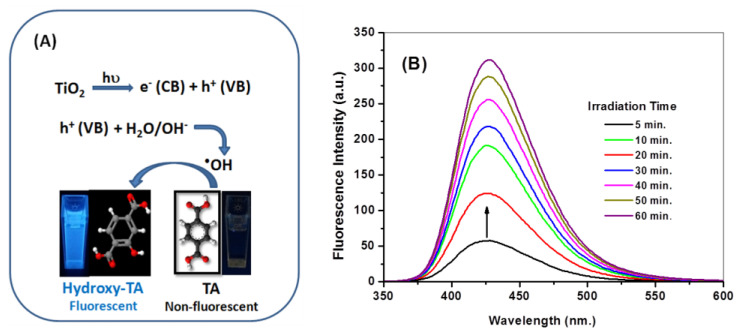
Diagrammatic illustration for the productivity of ^•^OH radicals via the suitable solar-driven catalytic photoexcitation of nano-titania (**A**). Fluorescence spectra of hydroxy terephthalate fluorophore (λ_ex_ = 315 nm) caused by ^•^OH trapping during the 60 min of the solar irradiation of nano-titania (**B**).

The productivity of ^•^OH radicals due to the solar irradiation of the nano-titania particles was precisely assayed via the photoluminescence process using a sodium terephthalate probe [41]. The mechanism is illustrated in the Figure 4A, where the solar irradiation results in photo-exciting the electrons, associating the valence band (VB) with the highly energetic conduction band (CB), and revealing hole (h^+^) formation to accommodate the valence band. The generated holes have the chance to interact with H_2_O molecules, producing the ^•^OH radicals that are precisely snared by the sodium terephthalate non-fluorescent probe, switching to the hydroxyl terephthalate fluorophore with a strong emission peak centered at λ_em_ = 426 nm (Figure 4B). As seen in Figure 4B, the remarkable increase in the fluorescent signal of hydroxyl terephthalate corresponds to the proportional increase in the ^•^OH radicals produced 60 min after solar irradiation [42]. A control test to investigate the influence of solar irradiating TA/NaOH solution in the absence of nano-titania reveals that no hydroxy terephthalate was generated [43].

### 2.5. Phenolics and Flavonoids of DSO and ECP–DSO

The total phenolic and flavonoid contents of DSO and ECP–DSO were quantified through the external standard method using Folin–Ciocalteu reagent as the gallic acid equivalent (GAE) and quercetin equivalent (QE), respectively. The total phenolic contents of DSO and ECP–DSO were calculated as 26.85 ± 0.50 and 17.4 ± 0.67 mg GAE/g extract, respectively. The total flavonoid contents of tested extracts were 11.41 ± 0.17 and 6.09 ± 0.12 mg quercetin/g tested extract, respectively. Remarkable variations in phenolics and flavonoids as a result of encapsulation, which can be ascribed to the processing technique and the behavior of DSO-active constituents during encapsulation, need further study. Flavonoids play a prime role in antioxidant activity, depending on their molecular structure and hydroxylation pattern [44].

### 2.6. Spectrofluorimetric Evaluation of the ^•^OH Radical Scavenging Potentiality of DSO and ECP–DSO

**Figure 5:**
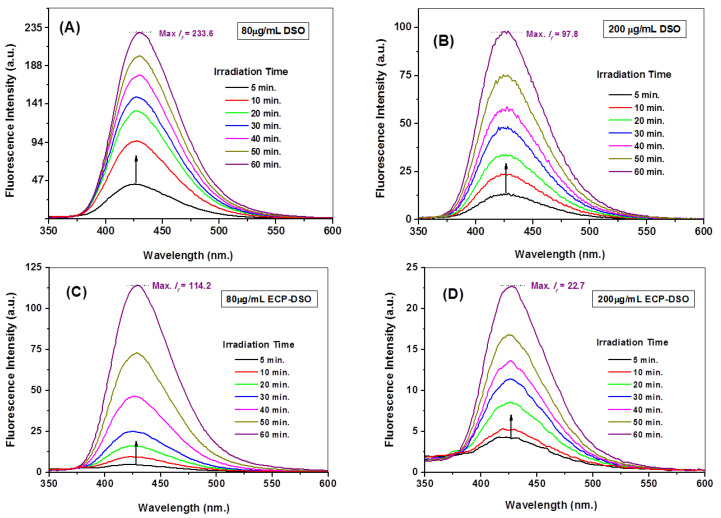
Temporal variations in fluorescence intensities of the formed hydroxyl terephthalate due to generated ^•^OH radicals via Solar-irradiated nanotitania in presence of (**A**) 80 μg/mL DSO; (**B**) 200 μg/mL DSO; (**C**) 80 μg/mL ECP-DSO; (**D**) 200 μg/mL ECP-DSO.

**Figure 6:**
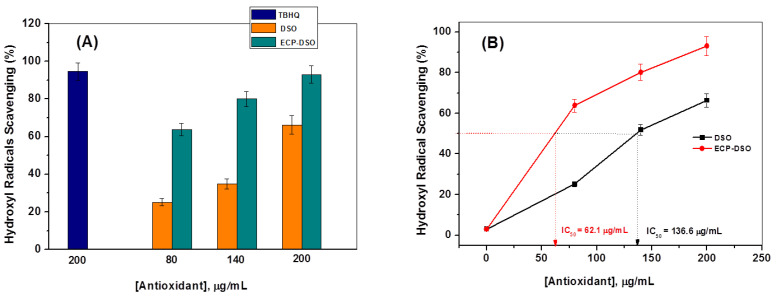
Hydroxyl radical scavenging (%) at different concentrations (80, 140, and 200 μg/mL) of DSO and ECP–DSO in comparison to the TBHQ antioxidant (200 μg/mL) (**A**). Hydroxyl radical scavenging in terms of IC50 for DSO and ECP–DSO (**B**).

The spectrofluorimetric assay detailed in Section 2.4 was repeated at two time intervals at three concentrations (80, 140, and 200 μg/mL) of either DSO or ECP–DSO samples that were kept on the shelf for five months at ambient conditions to test their potentialities towards ^•^OH radical scavenging and addressing the impact of encapsulation. At the same time, it is most important to examine the influence of the maltodextrin encapsulating material at the maximum added concentration of antioxidant material (200 μg/mL) on the fluorescence intensity of the fluorescent hydroxy terephthalate. The fluorescence intensities collected for hydroxyl terephthalate are negligibly influenced by the added encapsulating materials free from the DSO extract. This illustrates the absence of an interfering effect by the encapsulating materials on the fluorescence measurements and confirms the preciseness of the developed fluorimetric probing technique of ^•^OH radicals. Therefore, the time-dependent variation in the intensity of the fluorescence of hydroxyl terephthalate fluorophore 60 min after solar irradiating nano-titania in the presence of 80 and 200 μg/mL of DSO or ECP–DSO is depicted in Figure 5. As shown, the fluorescent signal (λ_em_ = 426 nm) distinguishing the ^•^OH radical trapping displayed a noticeable decrease in intensity at different grades. By adding 80 μg/mL of DSO or ECP–DSO, the max. *I_f_* deceases from 312 a.u. in the blank test to 233.6 and 114.2 a.u., corresponding to 25.12 and 63.39% ^•^OH scavenging, respectively. The amount of 200 μg/mL as the highest concentration of DSO or ECP–DSO strongly reduces the max. *I_f_* to 97.8 and 22.7 a.u., corresponding to 68.65 and 92.72% ^•^OH scavenging, respectively. Interestingly, the preliminary estimated ^•^OH scavenging activity when adding 200 μg/mL of either freshly extracted DSO or freshly encapsulated DSO revealed an initially higher ^•^OH percentage due to fresh DSO at 97.8% and 93.2% for fresh ECP–DSO. This tentatively suggests the merit of the encapsulation process. Figure 6A and Table 1 summarize the evaluated values of the scavenging percentage of ^•^OH radicals due to different concentrations of DSO or ECP–DSO in comparison to the estimated efficacy of ^•^OH scavenging at 200 μg/mL of the synthetic antioxidant (TBHQ).

Further comparative evaluation for DSO or ECP–DSO in ^•^OH radical scavenging was realized by estimating the IC50 values. IC50 refers to the sample concentration expressed in μg/mL, at which the inhibition was 50%. As shown in Figure 6B, the IC50 of DSO and ECP–DSO is 136.6 and 62.1 µg/mL, respectively. This decidedly demonstrates the supremacy of DSO in its encapsulated formulation over DSO in its pure form five months after extraction in terms of ^•^OH radical scavenging.

**Figure 7:**
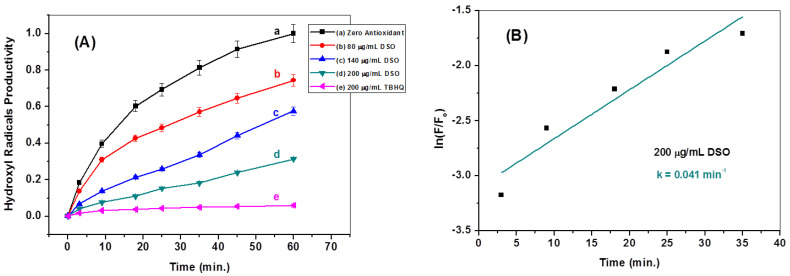
The kinetics of breeding ^•^OH radicals in the absence (line a) and the presence of different concentrations of DSO (lines b, c, and d) in comparison to TBHQ (line e) (**A**). Estimation of first-order rate constant in generating ^•^OH radicals in the presence of DSO (200 μg/mL) (**B**).

**Figure 8:**
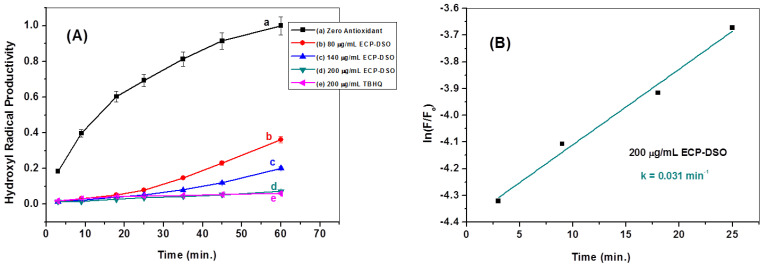
The kinetics of generating ^•^OH radicals in the absence (line a) and the presence of different concentrations of ECP–DSO (lines b, c, and d) in comparison to TBHQ (line e) (**A**). Estimation of first-order rate constant in generating ^•^OH radicals in the presence of ECP–DSO (200 μg/mL) (**B**).

Moreover, kinetic studies were employed to distinguish the efficacies of ^•^OH radical scavenging for DSO and ECP–DSO. The kinetics of generating ^•^OH radicals in the absence and presence of different doses of DSO and ECP–DSO, in addition to the synthetic antioxidant (TBHQ), are compared in Figure 7 and Figure 8. As illustrated, the increasing concentration of DSO gradually decreases the kinetics for generating ^•^OH radicals, while the potent declination of ^•^OH radicals’ productivity is achieved even when adding the lowest dose of ECP–DSO. The estimated rate constant for producing ^•^OH radicals in the blank experiment in the absence of an antioxidant was found to be 0.051 min^−1^, demonstrating that in the presence of 200 µg/mL of DSO and ECP–DSO, it decreased to 0.041 and 0.031 min^−1^, respectively, versus 0.028 min^−1^ when adding the same concentration of TBHQ (Table 1). This further highlights the benefit of encapsulation in preserving the activity of antioxidant ingredients, even after five months of encapsulation. As an additional benefit to its fascinating antioxidant performance, ECP–DSO is nearly as effective as the synthetic antioxidant TBHQ.

Contemplating the aforementioned results reveals the following points: (i) after five months of shelf-life, ECP–DSO preserves the efficacy of ^•^OH radical scavenging, while pure DSO loses considerable percentages of its ^•^OH scavenging capabilities; (ii) ECP–DSO exhibits considerable efficacy in scavenging ^•^OH radicals even at very low concentration (80 μg/mL); and (iii) when adding 200 μg/mL of ECP–DSO or the synthetic antioxidant (TBHQ), insignificant differences in ^•^OH radical scavenging activity are estimated, highlighting the prominence of ECP–DSO as a potent source of natural antioxidants that is a safe and promising alternative to synthetic antioxidant.

## 3. Materials and Methods

### 3.1. Materials

Dried dates (Sukkari) were collected from the local market in Sakaka City, Aljouf (KSA) as the supply for date seeds. Analytical grade reagents obtained from Sigma Aldrich Chemical Co. (St. Louis, Mo, USA), namely titanium dioxide (TiO_2_), benzyl alcohol, sodium hydroxide, terephthalic acid, ethanol, and tert-butylhydroquinone (TBHQ), were used. Deionized (DI) water was utilized to prepare all the working aqueous solutions.

### 3.2. Methods

#### 3.2.1. Preparation of Dried Seed Powder

The seeds were cleaned, washed to eliminate the peels, sun dried (4 days), and then manually crushed (grinding to fine particle size) using a heavy-duty mechanical grinder. Thereafter, the ground date seed powder was sieved through a 63 mesh in order to obtain appropriate particle sizes. The powdered seeds were kept in a plastic bottle and stored in a refrigerator for additional analysis.

#### 3.2.2. Date Seed Oil (DSO) Extraction

For oil extraction, a definite dried seed powder quantity (25 g) was placed into a Soxhlet apparatus (B-811, BUCHI, Essen, Germany) and n-hexane was used as the extraction solvent (400 mL). Depending on the solvent boiling point, the heating rate was adjusted. DSO extraction was performed (6 h) and then the obtained oil was cooled, flushed with a nitrogen stream, and the weight was calculated as the extraction yield. The DSO samples were stored (−20 °C) for further analysis and characterization.

#### 3.2.3. Gas Chromatography–Mass Spectrometry (GC/MS) of Date Seed Oil

Gas chromatography (GC) equipment (Shimadzu-QP2020, Japan) was employed to assess the content of the DSO fatty acids. The fitted GC had a flame ionization detector (FID) in the presence of carrier gas (helium). The date seed oil fatty acid composition is given in Table 2.

#### 3.2.4. Date Seed Oil Encapsulation

Date seed oil (DSO) was emulsified and then encapsulated using a Mini Spray Dryer (B-290, BÜCHI, Flawil, Switzerland) as previously described [45,46]. In a typical encapsulation method, 0.5 g of DSO was added to a 50 mL aqueous solution with maltodextrin and gum Arabic as wall materials (20% total solids) in a specific composition (maltodextrin:gum Arabic (80:20)), where the maltodextrin with dextrose equivalent (DE) range (16.5–19.5) was chosen for good water solubility, bland flavor, low moisture content during encapsulation, and low production costs [47,48]. For 1 min, the solution was continuously stirred with a magnetic stirrer at 150 rpm at room temperature (23 ± 2 °C). Spray drying was accomplished using compressed air at an 8 bar pressure, with the inlet air temperature justified at 130 °C.

#### 3.2.5. Measurement of Total Phenolics and Flavonoids

The total phenolic content (μg/g gallic acid equivalents) (GAE) and flavonoids (μg quercetin/g) (QE) of the tested extracts were spectrophotometrically determined at 700 nm using standard aluminum chloride and Folin–Ciocalteu reagent, respectively [49,50]. The amount of absorbed light is proportional to the number of oxidants present.

#### 3.2.6. Addressing the Average Crystallite Size and Band Gap Characteristics of the Nano-Titania

The crystallized size (D) of the utilized nano-titania was estimated via the Debye–Scherrer equation:

D = K λ/β cosθ (1)

where the Scherrer’s constant (K) had a value of 0.89; the X-ray wavelength (λ) had a value of λ = 1.54056 Å; θ is the Bragg angle; and β refers to the peak full width at the half maxima.

Moreover, the Kubelka–Munk Equation (2) addresses the band gap of the nano-titania:

(αhυ) = *A* (hυ − E_g_)^1/2^ (2)

where α is the extinction coefficient, h is Planck’s constant, *A* refers to an absorption constant, υ is the frequency, and E_g_ is the energy gap in eV.

#### 3.2.7. Generating Hydroxyl Radicals (^•^OH) via Solar Irradiation of TiO_2_-NPs and Spectrofluorimetric Monitoring

By subjecting nano-titania (8.0 mg), which was suspended in 50 mL of sodium terephthalate developed by mixing 5 × 10^−3^ mol L^−1^ of terephthalic acid to 1.0 × 10^−2^ mol L^−1^ NaOH, to solar irradiation of 3.83 × 10^4^ Lux (digitally measured using a PeakTech^®^ multi tester, Germany), hydroxyl radicals (^•^OH) were photocatalytically produced then assayed fluorimetrically. At a specific time point, 4.0 mL of the irradiated suspension was removed and the nano-titania particles were separated where the fluorescence spectrum was measured at λ_ex_ = 315 nm. The fluorescent signal characteristic for generating ^•^OH radicals is recorded at λ_em_ = 426 nm, which is indicative of the formation of hydroxyl terephthalate fluorophore.

#### 3.2.8. The Activity of DSO and ECP–DSO in Scavenging ^•^OH Radicals

The fluorimetric trapping and assessment protocol of ^•^OH radicals mentioned in Section 3.2.7 was developed to examine the efficacy of DSO and ECP–DSO in ^•^OH radical scavenging. Typically, in presence of three concentrations (80, 140, and 200 μg/mL) of DSO or ECP–DSO, the productive mixture of ^•^OH radicals was employed to begin the solar irradiation process.

The percentage of ^•^OH radical scavenging was evaluated using Equation (3):

(3) $$\mathrm{Scavenging}\;\mathrm{activity}\;(\%)=\left[\frac{\left(F_{0}-{\;F}_{\mathrm{anti}}\right)}{F_{0}}\right]\times 100$$

where F_o_ and F_anti_ are the maximum fluorescence intensities at λ_em_ 426 nm for the formed hydroxyl terephthalate fluorophore, respectively, in the absence and presence of examined antioxidants, while at the same time illuminating the productive solution of ^•^OH radicals.

Additionally, the IC50 value of the concentration of the antioxidant material required to specifically inhibit 50% of free ^•^OH radicals activity is determined. The IC50 value provides important information about the potency of an antioxidant material. A lower IC50 value indicates that less amount of the tested antioxidant is needed to achieve significant ^•^OH radical scavenging activity when the potency of DSO and ECP–DSO in scavenging ^•^OH radicals is compared.

According to the developed fluorimetric assay of ^•^OH radical scavenging and the methodology followed in Section 3.2.7, the IC50 is determined by the absence and presence of different concentrations through DSO or ECP–DSO. As the concentration of antioxidant material increases, more ^•^OH radicals are scavenged, resulting in a decrease in probe fluorescence at λ_em_ 426 nm. The IC50 value was then calculated as the concentration of DSO or ECP–DSO antioxidants required to reduce ^•^OH radical activity by 50%.

Moreover, on the basis of temporal variations of fluorescence intensities, fluorescent-dependent kinetics were developed in order to estimate the rates of scavenging ^•^OH radicals in the presence of the examined antioxidants, as shown in Equation (4):

ln(F/F_o_) = a + kt (4)

where F and F_o_ are, respectively, the fluorescence intensity (λ_em_ = 426 nm) for the formed hydroxy terephthalate fluorophore in presence and the absence of examined antioxidants at time (t) during the solar irradiation of the ^•^OH radicals’ generative system.

### 3.3. Instruments

The Agilent Cary 60 spectrophotometer and the Agilent Cary Eciplex spectrofluorimeter were used to record the UV–Vis absorption and emission spectra. The measurement of powdered XRD was found using the Maxima–X (D/Max2500VB2+/Pc) X-ray diffractometer (Shimadzu Company, Kyoto, Japan) with an X-ray wavelength Cu detector. Electron microscopy scanning (JEOL, JEM-2100, Japan) was used to provide the sample TEM imaging.

### 3.4. Statistical Analysis

SPSS software (version 16) was used to accomplish statistical analyses. The data were collected in triplicate and presented as (mean ± SD) and evaluated using Student’s *t*-test and the analyses of variance, where statistically significant differences were considered to be *p* < 0.05 as the means.

## 4. Conclusions

This work provides, for the first time, a sensitive and selective avenue to address the ^•^OH radical scavenging efficacy of date seed oil (DSO) in its pure and encapsulated formulations (ECP–DSO) both when fresh and 5 months after extraction and encapsulation. The developed approach employed the solar irradiation of nano-titania particles suspended in non-fluorescent sodium terephthalate to produce ^•^OH radicals that were selectively trapped by the terephthalate probe. Accordingly, fluorescence was sensitively performed using terephthalate hydroxylation with direct proportionality between fluorescence intensity and the amount of generated ^•^OH radicals with high precision. The percentage of ^•^OH scavenging reveals estimates of 25.12 and 63.39%, when adding 80 μg/mL of DSO or ECP–DSO, respectively, to the fluorimetric probing system that increased to 68.65 and 92.72% when adding 200 μg/mL of DSO or ECP–DSO, respectively. Moreover, the IC50 of DSO and ECP–DSO are 136.6 and 62.1 µg/mL, respectively. Furthermore, the estimated rate constant for producing ^•^OH radicals in the blank experiment (in the absence of antioxidant) was found to be 0.051 min^−1^, which decreases in the presence of 200 µg/mL of DSO or ECP–DSO to 0.041 and 0.031 min^−1^, respectively, versus 0.028 min^−1^ when adding the same concentration of TBHQ. Therefore, the developed encapsulation markedly preserves the potentiality of the DSO natural antioxidant even five months after extraction, which is, interestingly, close to the efficacy of the TBHQ synthetic antioxidant. Moreover, the selective fluorimetric assay provided for ^•^OH radicals paves the way for providing in-depth interpretations of antioxidant natural medicines with insights into the mechanisms of hydroxyl radical-damaging biology.

## Figures and Tables

**Table 1:** The estimates of hydroxyl radical scavenging (%) and the first-order rate constant for generating hydroxyl radicals at different doses of date seed oil (DSO) and encapsulated date seed oil (DSO–ECP) in comparison to TBHQ.

Sample	Concentration (μg/mL)	^•^OH Radical Scavenging (%) ± SD	Rate of ^•^OH Radical Productivity (min^−1^) ± SD	R^2^
DSO *	80	25.12 ± 0.28	--	--
	140	34.83 ± 0.35	--	--
	200	68.65 ± 0.22	0.041± 0.003	0.91
DSO-ECP *	80	63.39 ± 0.46	--	--
	140	80.11 ± 0.19	--	--
	200	92.71 ± 0.25	0.031 ± 0.002	0.98
TBHQ	200	94.60 ± 0.29	0.028 ± 0.001	0.93

* The samples are examined for hydroxyl radical scavenging after five months after extraction and encapsulation.

**Table 2:** Fatty acid composition, total phenolic content, and peroxide index of the date seed oil (DSO).

Date Seed Oil Content (% DW *) = 5.78		
Fatty Acids		(%)
Lauric	C12:0	15.17 ± 0.19
Myristic	C14:0	9.21 ± 0.11
Palmitic acid	C16:0	7.44 ± 0.20
Stearic acid	C18:0	6.36 ± 0.24
Arachidic (Eicosanoic)	C20:0	1.3 ± 0.12
Oleic acid	C18:1	46.32 ± 0.38
Linoleic acid	C18:2	13.03 ± 0.18
α-Linolenic acid	C18:3	1.15 ± 0.10
Total saturated FAs	SFA **	39.48 ± 0.32
Total Polyunsaturated FAs	PUFA ***	57.62 ± 0.41
Acid Value (AV) mg KOH/g DSO		0.59
Peroxide index (meq/kg DSO)		2.18
Total phenolic content (mg GAE/g)		26.85 ± 0.50
Flavonoids (mg QE/g)		11.41 ± 0.17

* DW: dry weight basis; ** SFA: saturated fatty acids; *** PUFA: polyunsaturated fatty acids.

## Data Availability

Not applicable.

## Sample Availability

Not available.
